# Antagomirs Targeting MiroRNA-134 Attenuates Epilepsy in Rats through Regulation of Oxidative Stress, Mitochondrial Functions and Autophagy

**DOI:** 10.3389/fphar.2017.00524

**Published:** 2017-08-08

**Authors:** Jiahang Sun, Xiaoying Gao, Dawei Meng, Yang Xu, Xichun Wang, Xin Gu, Mian Guo, Xiaodong Shao, Hongwen Yan, Chuanlu Jiang, Yongri Zheng

**Affiliations:** ^1^Department of Neurosurgery, The Second Affiliated Hospital of Harbin Medical University Harbin, China; ^2^Department of Anesthesiology, The Fourth Affiliated Hospital of Harbin Medical University Harbin, China; ^3^Department of Neurosurgery, China Medical University Aviation General Hospital Beijing, China; ^4^Department of Urology, Harbin Medical University Cancer Hospital Harbin, China; ^5^Department of Neurosurgery, Heilongjiang Provincial Hospital Harbin, China; ^6^Department of Head and Neck Surgery, Harbin Medical University Cancer Hospital Harbin, China; ^7^School of Basic Medical Sciences, Harbin Medical University Harbin, China; ^8^Department of Pediatric Hematology, Peking University International Hospital Beijing, China

**Keywords:** epilepsy, miR-134, oxidative stress, mitochondrial functions, autophagy

## Abstract

The effects of the existing anti-epileptic drugs are unsatisfactory to almost one third of epileptic patients. MiR-134 antagomirs prevent pilocarpine-induced status epilepticus. In this study, a lithium chloride-pilocarpine-induced status epilepticus model was established and treated with intracerebroventricular injection of antagomirs targeting miR-134 (Ant-134). The Ant-134 treatment significantly improved the performance of rats in Morris water maze tests, inhibited mossy fiber sprouting in the dentate gyrus, and increased the survival neurons in the hippocampal CA1 region. Silencing of miR-134 remarkably decreased malonaldehyde and 4-hydroxynonenal levels and increased superoxide dismutase activity in the hippocampus. The Ant-134 treatment also significantly increased the production of ATP and the activities of mitochondrial respiratory enzyme complexes and significantly decreased the reactive oxygen species generation in the hippocampus compared with the status epilepticus rats. Finally, the Ant-134 treatment remarkably downregulated the hippocampal expressions of autophagy-associated proteins Atg5, beclin-1 and light chain 3B. In conclusion, Ant-134 attenuates epilepsy via inhibiting oxidative stress, improving mitochondrial functions and regulating autophagy in the hippocampus.

## Introduction

Epilepsy is a serious chronic neurological disorder characterized by chronic and spontaneous seizures, and subjected more than 65 million people worldwide into both physical distress and psychological stress (Moshe et al., 2015). Although epilepsy has been extensively treated by anti-epileptic drugs, the effects are unsatisfactory to almost one-third of epileptic patients. Therefore, it is urgent to investigate novel treating targets and develop novel anti-epileptic drugs.

Mitochondria are important subcellular organelles involved in several key cell functions, and are usually known as energy producers for their roles in generating the majority of adenosine triphosphate (ATP). Mitochondria also participate in reactive oxygen species (ROS) generation, apoptosis and calcium homeostasis (Simeone et al., 2014). Mitochondrial dysfunction has been demonstrated in epilepsy patients and animals (Kunz et al., 2000; Chuang et al., 2004; Sleven et al., 2006; Lee et al., 2008; Azakli et al., 2013). Although the views about the role of energy supply deficiency caused by mitochondrial dysfunction in the pathological mechanisms are controversial (Kann et al., 2005; Kudin et al., 2009; Folbergrova and Kunz, 2012), it is definite that the increased neuronal excitability is related to dysfunction of mitochondrial oxidative phosphorylation. Excessive ROS generation is more important than abnormal energy supply (Jacobson et al., 2005). Oxidative stress, a key factor in the pathogenesis of epilepsy, would injure the mitochondrial respiratory chain and induce excessive ROS production. The accumulation of ROS can depress the activities of mitochondrial respiratory enzyme complexes and result in cell death in the epileptic area (Frantseva et al., 2000). In addition, attenuation of oxidative stress has been proved to protect the hippocampus from abnormal mossy fibers sprouting (MFS) (Baluchnejadmojarad and Roghani, 2013; Dariani et al., 2013). Therefore, targeting oxidative stress may benefit epileptic treatment.

Autophagy is a self-defensive process that protects survival against stress or nutrient deficiency (Gallagher et al., 2016). Under normal conditions, the autophagic system is suppressed to a basal level. Under pathological conditions, however, cytoplasmic components including damaged organelles and misfolded proteins are delivered into double-membrane autophagosomes, which then fuse with lysosomes, inducing autophagic degradation. Some studies have revealed the link between autophagy and epilepsy that autophagy promotes genetic epilepsy while epilepsy in turn induces autophagy (Gan et al., 2015). Our study focused on the epilepsy-induced autophagy because our animal model was induced pharmacologically, not genetically. ATP exhaust and ROS over-production, which participate in the pathological mechanisms of epilepsy, are important initiators of autophagy (Codogno and Meijer, 2005; Cao et al., 2009). The anti-epilepsy drugs that can inhibit oxidative stress in the epileptic areas may also affect the autophagic system.

Increasing evidence supports the important role of miRNAs, a family of small non-code RNA, in the pathogenesis of various diseases including epilepsy (Ambros, 2004; Henshall et al., 2016). In the brain, miR-134 is localized in the synapto-dendritic compartment of hippocampal neurons (Schratt et al., 2006). Studies reveal miR-134 is involved in the control of synaptic protein synthesis and plasticity, and thus in the regulation of learning and memory (Gao et al., 2010; Bicker et al., 2013). As reported, the hippocampal miR-134 level is significantly downregulated in epileptic rat and children (Peng et al., 2013), while silencing of miR-134 could delay seizure onset, and reduce seizure severity, Racine score and mortality of epilepsy animals (Jimenez-Mateos et al., 2012; Jimenez-Mateos et al., 2015). Mechanically, miR-134 blockage could reduce the density of pyramidal neuron spine in the CA3 region, enlarge its volume and decrease neuronal death. It is demonstrated that miR-134 could regulate a mitochondrial-apoptosis-associated protein Bcl-2 (Huang et al., 2015; Shao et al., 2015), indicating miR-134 may participate in the regulation of mitochondrial functions, which has not been reported yet. The aims of the present study were to investigate the possible effect of miR-134 on oxidative stress control and mitochondrial function maintenance in the hippocampus of epileptic rats, and to observe the changes of autophagy-associated proteins.

## Materials and Methods

### Animals

Male Sprague–Dawley (SD) rats (180–220 g) were obtained from the animal center at the Second Affiliated Hospital of Harbin Medical University (Harbin, China). All the animals were housed in 50–60% humidity and kept on a 12-h light/dark cycle with free access to food and water. All experimental procedures were approved by the Ethics Committee of the Second Affiliated Hospital of Harbin Medical University.

### Epilepsy Induction

A total of 103 rats were randomly divided into four groups: control group (Con, *n* = 18), status epilepticus (SE) group (SE, *n* = 30), scramble antagomirs sequence group (Scr, *n* = 30), and antagomir targeting miR-134 group (Ant-134, *n* = 25). SE was induced as described by Ashhab et al. (2013). Briefly, the rats in SE, Scr and Ant-134 groups were intraperitoneally injected first with 125 mg/kg lithium chloride (LiCl) and 18–20 h later with 20 mg/kg pilocarpine hydrochloride (both Sigma, St. Louis, MO, United States). In addition, 30 min prior to pilocarpine treatment, the rats were administrated intraperitoneally with 1 mg/kg Scopolamine methyl bromide (Sigma) to reduce the peripheral cholinergic effects of pilocarpine. The control rats in received equal amounts of saline. The SE rats were screened following the classification of Racine (1972), as only stage IV or V were enrolled here. To control the seizure intensity and decrease mortality, we used intraperitoneal administration of 10 mg/kg diazepam (Sigma) to SE rats 90 min after the onset of SE. However, 12 SE rats, 11 Scr rats and 6 Ant-134 rats died. One Scr rat and 1 Ant-134 rat were randomly selected for some preliminary experiments.

### Intracerebroventricular Injections

Immediately after the SE model was established, the rats were anesthetized with 10% chloral hydrate, placed in a stereotaxic frame and inserted with a 23-gaged stainless-steel guide cannula into the bilateral ventricle through a hole drilled through the skull 4.4 mm below the top of the skull, 1.5 mm lateral and 0.8 mm posterior to the bregma. Rats were infused with 2 μL of 0.12 nM Ant-134 or a non-targeting scrambled version of the antagomir (Scr) (GenePharma, Shanghai, China) in artificial cerebrospinal fluid at a speed of 0.2 μL/min. The cannula was remained in the brain for additional 10 min. Four weeks after the injections, six rats in each group were randomly selected for Morris water maze (MWM) test and the other rats were sacrificed at day 3 for histological and biological analyses.

### Morris Water Maze

Morris water maze tests were carried out in a black cylindrical tank (150 cm in diameter and 40 cm in height) containing 24 cm depth of water (22 ± 2°C). The tank was divided into four quadrants, each with four starting points were at the edges. A circular platform (12 cm in diameter) was submerged 1 cm below the water. Visual cues were located on the wall center of each quadrant. The rats were sent to four tests per day for four consecutive days. In each test, a rat was placed facing the tank walls in the water randomly at one of the four starting points. The swimming paths and time of the rat were recorded by a camera placed above the maze center. If the rat failed to find the platform within 60 s, it was guided to the platform and allowed to stay there for 30 s and the latency was recorded as 60 s. In the probe test (day 5), the platform was removed, and the rat was placed at the starting point at the quadrant opposite to the platform quadrant and allowed to swim freely for 60 s. The percentage of time spent in the target quadrant was recorded.

### Nissl Staining

The brain tissues were fixed in 10% paraformaldehyde for 24 h, dehydrated in a series of ethanol solutions and embedded in paraffin. Then the paraffin blocks of the brain were sliced into 5-μm-thick sections, which were deparaffinized, rehydrated and stained with 0.5% cresyl violet at room temperature for 10 min. The stained sections were dehydrated, mounted, and the CA1 region of the hippocampus were examined under a BX51 light microscope (Olympus, Tokyo, Japan). The Nissl-positive cells from 3 randomly selected sections of each rat were counted by two technicians in a blinded manner.

### Timm’s Staining

Timm’s staining was performed using a commercial kit (Genmed Scientifics Inc., Wilmington, DE, United States) following the manufacture’s protocol. In each test, the rat was deeply anesthetized and perfused in a sulfide solution for 10 min. The brain was gently collected and immersed in the sulfide solution for 45 min at room temperature. After washing in deionized water, brain tissues were fixed in fixatives provided in the kit, embedded in paraffin and cut into sections following the standard protocol. Dewaxed brain tissue sections were stained with the working solution at room temperature in the dark for 80 min and washed in the cleaning solution for 5 min. The stained brain tissue sections were observed under a DP73 light microscopy (Olympus).

### Terminal deoxynucleotidyl transferase dUTP Nick End Eabeling (TUNEL) Staining

Dewaxed brain tissue sections were stained using an *in situ* cell death detection kit (Roche Diagnostics, Mannheim, Germany) according to the manufacturer’s instructions, followed by co-staining with hematoxylin (Solarbio Science & Technology, Co., Ltd., Beijing, China). TUNEL-positive cells from 3 randomly selected sections of each animal were counted under the BX51 light microscope by two technicians in a blinded manner.

### Oxidative Stress Assays

To assess the oxidative stress states in the hippocampus, we evaluated malonaldehyde (MDA) level, 4-hydroxynonenal (4-HNE) level and superoxide dismutase (SOD) activity using biochemical kits (Nanjing Jiancheng Bioengineering Institute, China) following the manufacture’s protocols. The hippocampus tissues were homogenized in a phosphate buffer solution on ice and freeze-thawed in liquid nitrogen three times. The homogenate was centrifuged at 10,000 *g* and 4°C for 10 min and then the supernatant was collected for tests. The total protein concentration was determined using a bicinchoninic acid (BCA) protein assay kit (Beyotime Institute of Biotechnology, Haimen, China).

### RNA Extraction and Quantitative Real-Time PCR

Total RNA was isolated from the hippocampus using a total RNA extraction kit (BioTeke Corporation, Beijing, China), according to the manufacturer’s protocol. RNA purity and concentration were determined using a NANO2000 spectrophotometer (Thermo Fisher Scientific, Waltham, MA, United States). Complementary DNA (cDNA) was reversely transcribed with an oligonucleotide primer using a super Moloney murine leukemia virus (M-MLV, BioTeke). qPCR was carried out in 20 μl of the reaction system containing 1 μl of cDNA, 10 μl of 2 × Power Taq PCR Master Mix (BioTeke) and SYBR Green (Solarbio), 0.5 μl of forward and reverse primer (10 μM, **Table 1**) dissolved in double-distilled water on an Exicycler 96 (Bioneer, Daejeon, Korea). All tests were conducted in triplicate. Copy numbers of RNA were quantified using the comparative ΔΔCt method, with β-actin or U6 as the internal control.

**Table 1:** Sequences of primers.

Genes	Sequence F (5′-3′)	Sequence R (5′-3′)	Size (bp)
mir-134	GACTGGCTGTGAC TGGTTGACC	GTGCAGGGTCC GAGGTATTC	63
U6	CTCGCTTCGGCA GCACA	AACGCTTCACGAATT TGCGT	94
Atg-5	AGTGGAGGCAACAG AACCC	TCCGACCACCGTCAC CTTA	250
LC3B	CACAGTCTTTGTAAG GGCGGTTCT	GGCTTGCTTTAGTT GGAAGTGG	145
Beclin-1	CAGCAGTTCAAAGA AGAGGTG	GAGGACACCCAAGC AAGAC	235
β-actin	GGAGATTACTGCCCT GGCTCCTAGC	GGCCGGACTCATCGT ACTCCTGCTT	155


### Western Blotting Analysis

Total protein from hippocampal tissues was extracted using radio- immunoprecipitation assay with phenylmethanesulfonyl fluoride and the protein concentration was determined using the BCA protein assay kit. A total of 40 μg proteins from each sample were loaded on 10% sodium dodecyl sulfate polyacrylamide gels and the target proteins were transferred to polyvinylidene difluoride membranes. In immunoblotting, membranes were blocked with 5% non-fat milk in Tris buffered saline, with Tween-20 (TBST) at room temperature for 1 h and incubated with Atg-5 antibody (1:400, BA3525-2, BOSTER Biological Technology CO. LTD., Huhan, China), light chain 3B (LC3B) antibody (1:500, BM4827, BOSTER), Beclin-1 antibody (1:400, BA3123-2, BOSTER), or β-actin antibody (1: 1000, sc-47778, Santa Cruz Biotechnology, Santa Cruz, CA, United States) at 4°C overnight. Then the membranes were incubated with a horseradish peroxidase-conjugated anti-rabbit or anti-mouse secondary antibody (1:5000, Beyotime) at 37°C for 45 min. Bands were visualized with enhanced chemiluminescence (7 Sea Pharmtech, Shanghai, China) and analyzed on a gel-pro-analyzer (Media Cybernetics, Bethesda, MD, United States).

### Statistical Analysis

Data are expressed as mean ± standard deviation (SD) and analyzed by one-way analysis of variance (ANOVA) followed by the Fisher’s least significant difference (LSD) test. All statistical analyses were performed on SPSS 19.0. Values of *P* <0.05 were considered statistical different.

## Results

### Ant-134 Injection Improves Performance of SE Rats in MWM Tests

**FIGURE 1:**
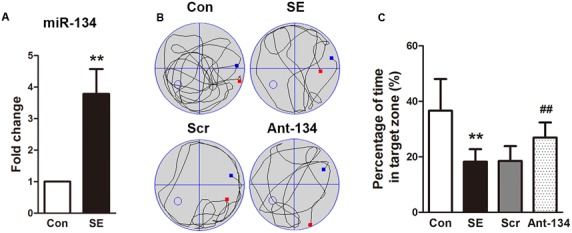
Effects of Ant-134 on spatial learning memory formation. **(A)** miR-134 expression was upregulated in the hippocampus of SE rats. **(B)** Tracings of typical swim patterns and **(C)** proportion of time spent in target quadrant during the probe trail. The small circle in the third quadrant indicates the position of the escape platform. Data are expressed as mean ± SD, *n* = 6. ^∗∗^*p* < 0.01, versus the control group, ^##^*p* < 0.01, versus the SE group.

We first detected the mRNA expression of miR-134 in hippocampal tissues of SE rats at day 3 after LiCl-pilocarpine injection. like previous studies (Jimenez-Mateos et al., 2012, 2015), real-time quantitative PCR analysis showed the hippocampal miR-134 level was significantly increased in the LiCl-pilocarpine-induced SE rats (**Figure 1A**).

To investigate the effect of Ant-134 on the hippocampal function, we tested performance in the MWM test, a typical measurement of hippocampus-dependent spatial memory. As expected, the SE rats spent significantly less time in searching the target quadrant compared to the control rats (**Figures 1B,C**). However, the Ant-134 rats spent markedly higher time in the target zone, which indicate that Ant-134 may benefit the hippocampal function in SE rats.

### Ant-134 Injection Inhibits MFS

**FIGURE 2:**
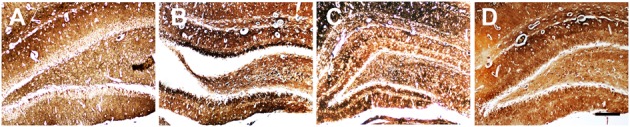
Timm’s staining of zinc-containing mossy fiber terminal fields in hippocampal CA3 region in the **(A)** control, **(B)** SE, **(C)** Scr and **(D)** Ant-134 groups. Scale bar: 100 μm.

Mossy fibers sprouting in the dentate gyrus of hippocampus, which accompanies epileptogenesis, was visualized using Timm’s staining to label the zinc-containing mossy fibers from granule cells. In the control rats, little sprouting was found in the dentate gyrus. In contrast, a dense band was observed in the SE rats, indicating the occurrence of aberrant MFS in the hippocampus, which is consistent with previous studies on various epilepsia models (Sharma et al., 2007; Kuo et al., 2008; Baluchnejadmojarad and Roghani, 2013). In the Ant-134 rats, the stained MFS in the dentate gyrus was dispersed and less intense (**Figure 2**), indicating Ant-134 can restrain LiCl-pilocarpine-induced MFS in rats.

### Ant-134 Injection Inhibits Neuronal Damage in the Hippocampus of SE Rats

**FIGURE 3:**
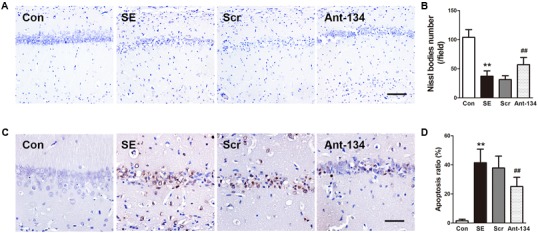
Silencing miR-134 attenuated neuronal death in hippocampus of SE rats. **(A)** Nissl staining of hippocampal neurons. **(B)** Quantification of Nissl body number. **(C)** TUNEL staining of hippocampal sections. **(D)** Quantification of TUNEL-positive cells. Scale bar: 100 μm in A and 50 μm in **(C)**. Data are expressed as mean ± SD, *n* = 6. ^∗∗^*p* < 0.01, versus the control group, ^##^*p* < 0.01, versus the SE group.

Neuronal loss and apoptosis in the CA1 region were assessed using Nissl and TUNEL staining 3 days after SE induction. Nissl staining showed the integrative neurons of the control rats were morphologically normal. On the contrary, the SE rats were found with dissolved neurons and loss of Nissl bodies, while Ant-134 rats were found with significantly improved survival and normalized morphology of neurons (**Figures 3A,B**). In line with the Nissl staining, the numbers of apoptotic cells in the CA1 region were very large in the SE rats, but were significantly lowered in Ant-134 rats (**Figures 3C,D**).

### Ant-134 Treatment Attenuates Oxidative Stress in the Hippocampus

**FIGURE 4:**
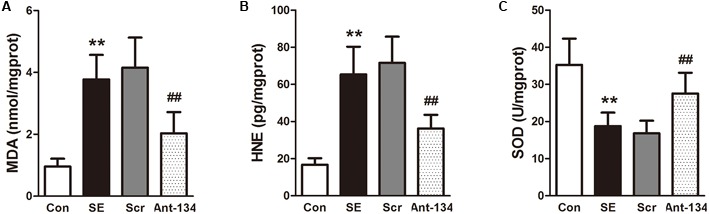
Effects of Ant-134 on oxidative stress in the hippocampus of the SE rats. Treatment of Ant-134 significantly decreased **(A)** MDA and **(B)** HNE production and increased **(C)** SOD activity. Data are expressed as mean ± SD, *n* = 6. ^∗∗^*p* < 0.01, versus the control group, ^##^*p* < 0.01, versus the SE group.

Peroxidation status in the hippocampus was evaluated by measuring the concentrations of MDA and 4-HNE, two lipid peroxidation markers. Results demonstrated that the hippocampal MDA and 4-HNE concentrations are dramatically higher in the SE rats, and are significantly reduced in Ant-134 rats (**Figures 4A,B**). The activity of hippocampal SOD is much lower in the SE rats than in the control rats, and is significantly enhanced in the Ant-134 rats (**Figure 4C**).

### Ant-134 Injection Improves Mitochondrial Functions in Hippocampus of SE Rats

**FIGURE 5:**
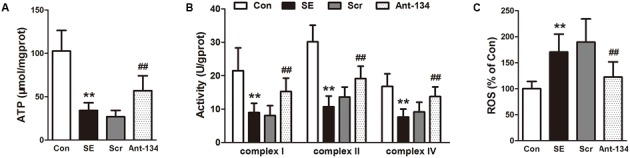
Effects of Ant-134 on mitochondrial function in hippocampus of the SE rats. Treatment of Ant-134 enhanced **(A)** ATP production and **(B)** activities of complex I, II, and IV and decreased **(C)** ROS production. Data are expressed as mean ± SD, *n* = 6. ^∗∗^*p* < 0.01, versus the control group, ^##^*p* < 0.01, versus the SE group.

The mitochondrial functions were evaluated by measuring ATP concentration, ROS production, and activities of mitochondrial respiratory chain complex. As illustrated in **Figure 5**, ATP concentration and the activities of complexes I, II, and IV are significantly reduced and ROS production is increased in the SE rats, which indicate the occurrence of mitochondrial dysfunction. Nevertheless, these changes were effectively restored by the silencing of miR-134 with Ant-134.

### Ant-134 Injection Normalizes Hippocampal Autophagy of SE Rats

**FIGURE 6:**
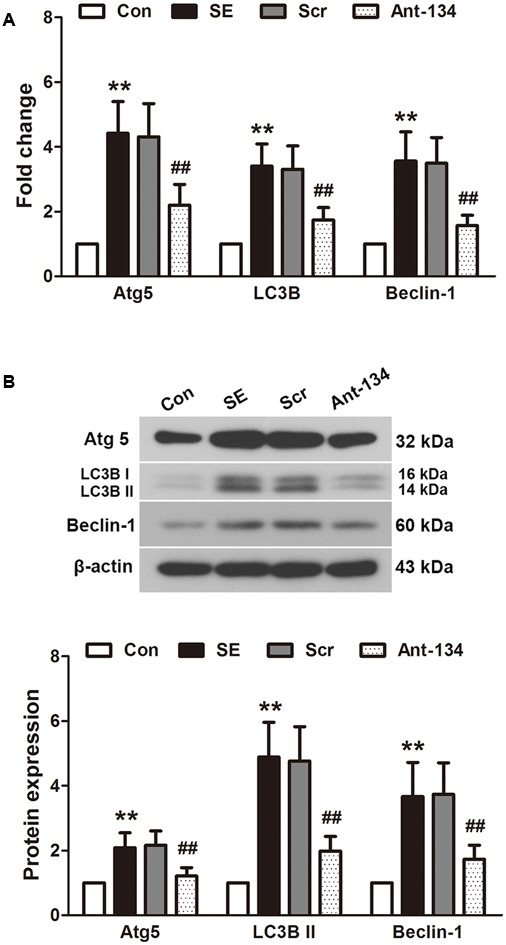
Effects of Ant-134 on autophagy in the hippocampus of SE rats. Treatment of Ant-134 downregulated **(A)** mRNA and **(B)** protein expressions of Atg5, LC3B and beclin-1. Data are expressed as mean ± SD, *n* = 6. ^∗∗^*p* < 0.01, versus the control group, ^##^*p* < 0.01, versus the SE group.

The expression of autophagy markers Atg5, LC3B II and beclin 1 were determined using quantitative PCR (**Figure 6A**) and Western blot (**Figure 6B**). The mRNA and protein expressions of these three markers are all significantly higher in the SE rats compared with the control rats. The mRNA expressions are in parallel with the protein expressions. These altered mRNA and protein levels can be reversed by the Ant-134 injection.

## Discussion

The present study demonstrates the neuroprotection of Ant-134 against LiCl-pilocarpine-induced epilepsy via improving hippocampal function. The mechanisms may be associated with the attenuation of oxidative stress and apoptosis, improvement of mitochondrial functions and recovery of autophagy in the hippocampus.

The temporal lobe is the most epileptogenic region in the brain, and temporal lobe epilepsy (TLE) is the most common epilepsy in humans (Bertram, 2009). Hippocampal dysfunction resulting from neuronal hyperexcitability is a prominent characteristic of TLE patients and animals (Dudek et al., 1994; Glass and Dragunow, 1995; Zhong et al., 2016). Granule cells, which play a key role in excitatory conduction, are the principal cell type in the dentate gyrus, an important part of the hippocampal formation in the temporal lobe. Under normal conditions, granule cells are generated in the hilus or subgranular zone and migrate to the granule cell layer (Scharfman and Pierce, 2012). They participate in learning and memory by projecting with mossy fibers to the hippocampal CA 3 region (Scott and Holmes, 2012). TLE animals and patients were found with ectopic granule cells, including increased number and abnormal position of cells and aberrant MFS (Dudek et al., 1994; Sugaya et al., 2010). These ectopic granule cells form a more complex network, which receive excessive excitatory input and result in the hyperexcitable system. Meanwhile, they impact the learning and memory, two important functions of hippocampus. In the present study, the phenomena observed in the LiCl-pilocarpine induced SE rats are similar to previous studies, including the impaired spatial learning and memory function and aberrant MFS. In addition, the death of neurons in the CA1 region also increased in SE rats, which is consistent with previous findings (Chen et al., 2016). Silencing miR-134 using Ant-134 attenuated MFS in the dentate gyrus, inhibited neuron death in the CA1 region and improved the cognitive function of SE rats. The effect of Ant-134 on evoked seizures suppressing (Jimenez-Mateos et al., 2012) as well as its effect on CA3 neurons (Jimenez-Mateos et al., 2015) have been reported. Here we extend the knowledge of miR-134 silencing to its effects on dentate gyrus and CA1 neurons but we did not reveal the detailed molecular targets of miR-134, which will be investigated in the future.

Oxidative stress and ROS play important roles in the pathogenesis of epilepsy. On one hand, oxidative stress impairs the mitochondrial respiratory chain, causes overproduction of free radicals and damages the antioxidant enzyme system. On the other hand, these excessive free radicals and the inhibited antioxidant enzymes exacerbate the oxidative stress responses, forming a vicious cycle (Frantseva et al., 2000; Malinska et al., 2010). Finally, oxidative stress and mitochondrial dysfunction disrupt cellular homeostasis, which induces neuronal hyperexcitability and neuronal death in epilepsy (Chang and Yu, 2010). In consistence with this knowledge, the present study shows a significant increase in oxidative stress in the hippocampus challenged with LiCl-pilocarpine, as evidenced by the markedly increase of MDA and HNE levels and the decrease of SOD activity. MDA and HNE are usually used as oxidative stress markers. Particularly, HNE can bind to mitochondrial proteins and alter their functions (Barrera et al., 2016), which in turn further promote mitochondrial dysfunction. The present study demonstrates that silencing of miR-134 significantly attenuates oxidative stress in the hippocampus, suggesting miR-134 may contribute to the oxidative stress in the development of epilepsy. Additionally, the decrease of ATP production and activities of mitochondrial respiratory enzyme complexes and the increase of ROS production suggest the occurrence of mitochondrial dysfunction. Mitochondria are a primary source of ROS, which renders them vulnerable to oxidative damage (Waldbaum and Patel, 2010). Animal and human TLE specimens prove the occurrence of mitochondrial dysfunction during epilepsy (Kunz et al., 2000; Chuang et al., 2004; Gao et al., 2007; Vielhaber et al., 2008; Waldbaum et al., 2010). The present study indicates silencing of miR-134 protects the hippocampus from oxidative damage and improves mitochondrial functions. Previous research reveals the anti-oxidation ability of Ant-134 in H_2_O_2_-induced retinal ganglion cells, and shows the target of miR-134 is the cyclic AMP-response element-binding protein (CREB) (Shao et al., 2015). However, it should be further studied whether the epileptic hippocampal miR-134 shares the same signaling pathway with the retinal miR-134.

Oxidative stress leads to autophagy in epilepsy (Cao et al., 2009; Li et al., 2015). In line with the previous study, we find the autophagic system is impaired in the hippocampus of LiCl-pilocarpine-induced rats, as evidenced by the upregulation of autophagy-associated proteins Atg5, LC3B II and beclin 1. Under normal conditions, autophagy participates in synaptic function regulation and protects cellular homeostasis (Gan et al., 2015; Shen et al., 2015). In our SE model, the oxidative stress and ATP exhaust resulted in abnormal autophagy, which may in turn further aggravate epilepsy (Gan et al., 2015). The abnormal autophagy can be restored by treatment with Ant-134 through the down-regulation of autophagy-associated proteins. However, we did not find any direct target of miR-134 in the autophagic system. This autophagy-regulating effect may be a result of oxidative stress inhibition.

## Conclusion

Antagomirs targeting miR-134 attenuates LiCl-pilocarpine-induced SE in rats via inhibiting oxidative stress and improving mitochondrial and autophagic functions in hippocampus. The effects of mitochondrial and autophagic regulation may be associated with its anti-oxidation ability. Our findings provide new evidence that antagomirs may be used for seizure suppression.

## Author Contributions

The experiments were performed by JS, XyG, DM, YX, XW, XG, MG, XS, and HY. The data were analyzed by JS and XyG. The manuscript was written by JS and XyG and revised by CJ and YZ. The study was designed and the funding was obtained by CJ and YZ.

## Conflict of Interest Statement

The authors declare that the research was conducted in the absence of any commercial or financial relationships that could be construed as a potential conflict of interest.
